# Global Data Analysis Shows That Soil Nutrient Levels Dominate Foliar Nutrient Resorption Efficiency in Herbaceous Species

**DOI:** 10.3389/fpls.2018.01431

**Published:** 2018-09-26

**Authors:** Zhiqiang Wang, Zhexuan Fan, Qi Zhao, Mingcheng Wang, Jinzhi Ran, Heng Huang, Karl J. Niklas

**Affiliations:** ^1^The Institute for Advanced Study, Chengdu University, Chengdu, China; ^2^State Key Laboratory of Biocontrol, School of Life Sciences, Sun Yat-sen University, Guangzhou, China; ^3^Key Laboratory for Bio-Resources and Eco-Environment, College of Life Sciences, Sichuan University, Chengdu, China; ^4^State Key Laboratory of Grassland and Agro-Ecosystems, School of Life Sciences, Lanzhou University, Lanzhou, China; ^5^Department of Environmental Science, Policy, and Management, University of California, Berkeley, Berkeley, CA, United States; ^6^Plant Biology Section, School of Integrative Plant Science, Cornell University, Ithaca, NY, United States

**Keywords:** global scale, nutrient resorption, climatic factor, soil nutrients, herbaceous species

## Abstract

Nutrient resorption plays an important role in ecology because it has a profound effect on subsequent plant growth. However, our current knowledge about patterns of nutrient resorption, particularly among herbaceous species, at a global scale is still inadequate. Here, we present a meta-analysis using a global dataset of nitrogen (N) and phosphorus (P) resorption efficiency encompassing 227 perennial herbaceous species. This analysis shows that the N and P resorption efficiency (NRE and PRE, respectively), and N:P resorption ratios (NRE:PRE) across all herbaceous plant groups are 59.4, 67.5, and 0.89%, respectively. Across all species, NRE, PRE, and NRE:PRE, exhibited different patterns along climatic and soil nutrient gradients, i.e., NRE decreases with increasing mean annual precipitation (MAP) and soil N, PRE increases with aridity index (AI) but decreases with MAP and soil P, and NRE:PRE decreases with increasing potential evapotranspiration (PET), AI, and soil N:P. NRE, PRE, and NRE:PRE also differed in functional species group (graminoids vs. forbs). Soil nutrient level was the largest contributor to the total variations in NRE, PRE, and NRE:PRE, while climate and herbaceous types had relatively smaller effects on NRE, PRE, and NRE:PRE. Collectively, these trends can inform attempts to model biogeochemical cycling at a global scale.

## Introduction

Nutrient resorption (i.e., internal nutrient recycling) is recognized as one of the most important mechanisms in plant ecology because it permits plants to re-use nutrients directly and reduces their dependence on external nutrient supplies, especially in nutrient-poor environments (Aerts, 1996; Aerts and Chapin, 1999). This conservation mechanism can affect many ecological processes such as plant competition, nutrient uptake, reproduction, and carbon cycling (Killingbeck, 1996; Berg and McClaugherty, 2008; Richardson et al., 2008; Zhang et al., 2013).

Nitrogen (N) and phosphorus (P) are the main nutrients most frequently restricting plant growth and production globally (Chapin, 1980; Güsewell, 2004). N and P resorption efficiency are often presented as two important indices of internal nutrient recycling in plants. The resorption efficiency of N (NRE) and P (PRE) is defined as the proportional resorbed of N and P during leaf senescence: NRE or PRE = [(N or P in green leaves – N or P in senesced leaves)/N or P in green leaves] × 100% (Killingbeck, 1996; Kobe et al., 2005; Yuan and Chen, 2009). Thus, the resorption of N and P is of paramount importance to plant nutrient conservation (Killingbeck, 1996; Kobe et al., 2005).

Over the past decades, great progress has been made on understanding the interactions among foliar NRE and PRE and ambient climatic factors at the local (Wright and Westoby, 2003; Tully et al., 2013; Zhao et al., 2017), regional (Tang et al., 2013; Sun et al., 2016; Xu et al., 2017) and global levels (Kobe et al., 2005; Yuan and Chen, 2009; Vergutz et al., 2012). The foliar NRE, PRE and their ratios have been widely explored to indicate nutrient limitation (Güsewell, 2004; Richardson et al., 2008) and its response to environmental change (Yuan and Chen, 2009; Reed et al., 2012). Thus, a quantitative understanding the foliar nutrient resorption patterns of plants can offer insights into plant nutrient limitations, and possibly into the different responses of plants to multiple global climate changes and nutrient cycling process (Aerts and Chapin, 1999; Chapin et al., 2011).

Previous work has indicated that foliar N and P resorption is regulated by climatic factors and that they show distinct biogeographic patterns (Richardson et al., 2005; Yuan and Chen, 2009; Reed et al., 2012; Vergutz et al., 2012; Tang et al., 2013; Brant and Chen, 2015). In particular, most meta-analyses at a global or regional level seem to support the assumption that NRE and PRE should be related to latitude, climatic factors, and soil nutrient levels (Yuan and Chen, 2009; Vergutz et al., 2012; Tang et al., 2013), although these assumptions are somewhat controversial. For example, Yuan and Chen (2009) found that within different plant functional groups (tree, shrub, broadleaf, and conifer species), NRE and PRE have opposite trends with respect to MAT and MAP, i.e., NRE decreases with increasing MAT and MAP, whereas PRE increases with increasing MAT and MAP. These trends are consistent with the results reported by Tang et al. (2013) in Eastern China for woody plants. In contrast, Vergutz et al. (2012) reported that both NRE and PRE decrease with MAT and MAP at a global level and rejected the previous hypothesis that plants always display higher NRE and PRE in low-fertility soils. Moreover, the N:P resorption ratio (NRE:PRE) patterns in previous studies also show significant differences. For example, Reed et al. (2012) reported that NRE:PRE is correlated negatively with MAT and MAP at the global scale. However, Sun et al. (2016) found evidence that there is no significant relationship between NRE:PRE and MAT and MAP at regional scales.

These and other contradictory findings present an obstacle to modeling global biogeochemical cycling. In particular, most meta-analyses conducted at a global level have focused on nutrient resorption among woody species, with little or no concern about herbaceous plants (Vergutz et al., 2012). This gap in our knowledge is particularly important because grasses play a substantial role in a range of global-scale processes, including productivity and nutrient cycling. Consequently, an understanding of the nutrient-resorption characteristics of herbaceous species can have important implications (Hobbie, 1992; Knops et al., 2002; Zhou et al., 2006).

In this study, we assembled a global database from published studies to explore three important variables of interest to clarify the significant factors affecting herbaceous NRE, PRE, and NRE:PRE at a global scale: (1) variations in NRE, PRE, and NRE:PRE across a diverse spectrum herbaceous species, and (2) biogeographic patterns of foliar NRE, PRE, and NRE:PRE in herbaceous species and their relationship with climatic factors and respective soil nutrient levels. In addition, nutrient resorption strategies have been found to vary significantly among plant functional groups (Aerts, 1996; Han et al., 2005). Given this, we also attempted to test if there are differences in NRE, PRE, and NRE:PRE within functional species groups (graminoids vs. forbs).

## Materials and Methods

### Data Collection

**FIGURE 1:**
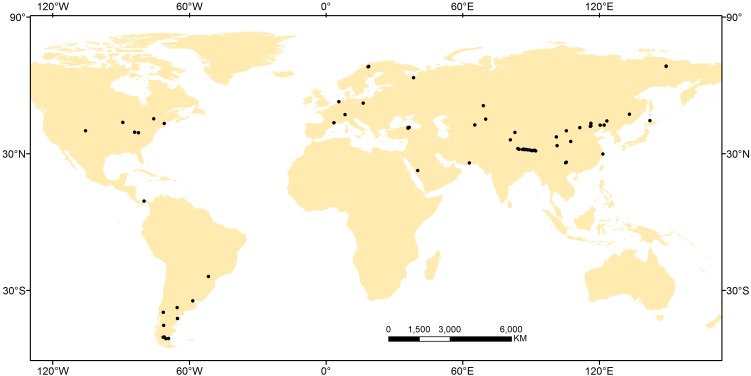
Distribution of the 66 sampling sites used in this study.

A global meta-analysis was conducted using published data for NRE and PRE (see **Supplementary Material**). To ensure data comparability, we compiled data from papers in which the authors specifically indicated that leaf litter samples came from newly fallen leaves that fell naturally or from freshly filled litter-traps, and green leaf samples came from fully expanded green leaves during the growing season. Given the leaf mass loss when leaf senesces (van Heerwaarden et al., 2003), we used the mass loss correction factor (MLCF), which was calculated from the percentage of leaf mass loss during senescence (Vergutz et al., 2012) to correct NRE and PRE, if green- and senesced-leaf mass loss were not available from the original study, i.e., NRE or PRE = [(N or P in green leaves – N or P in senesced leaves) × MLCF/N or P in green leaves] × 100%. The MLCF values were different: 0.731 for graminoid species and 0.64 for forb species (Kazakou et al., 2007; Vergutz et al., 2012). Further, we excluded data from leguminous plants (N-fixing species), annual plants, plants grown under greenhouse conditions, and from fertilized study plots. We used the Global Gazetteer Version 2.2^1^ and WorldClim 1.4 database^2^ to determine latitude, longitude, altitude, temperature, potential evapotranspiration (PET), aridity index (AI) and precipitation data (a global dataset with spatial resolution of *c*. 1 km^2^) if this information was missing in the original paper. In total, the database encompasses 227 perennial herbaceous species from 66 sites (**Figure 1** and **Supplementary Table S1**). Across this global data set, sites ranged from 0 to 4756 m in altitude, from −9 to 27°C in MAT, and from 7.3 to 4000 mm year^−1^ in MAP. Accordingly, the database broadly covers most of the range of MAT and MAP occupied by the majority of herbaceous species and thus permits a detailed global level of analysis not previously possible. Based on the Global Gridded surfaces of Selected Soil characteristics (Global Soil Data Task Group, 2000) and Global Gridded Soil Phosphorus Distribution Maps (Yang et al., 2014), we extracted the total N and P density in the top 50 cm of soil. Total N and P density were used in this study rather than plant-available forms because it is almost impossible to acquire soil N and P availability data which were missing in the vast majority of literatures we collected. Additionally, total soil nutrient content should be at least partially correlated with soil available nutrient status (Bridgham et al., 1995) and has been used widely in large scale ecological studies (Rejmánková, 2005; Zhang et al., 2018).

### Data Analysis

Due to the differences in soil nutrient availability at different sites where mycorrhizal types may have effects on NRE, PRE, and NRE:PRE, we only compared in the study sites where these two functional groups co-occurred. The mean values of NRE, PRE, and NRE:PRE between graminoids and forbs were assessed using one-way analysis of variance (ANOVA) and least-significant difference (LSD) *post hoc* analyses when effects were significant. Data for NRE, PRE, and NRE:PRE were log_10_-transformed before analysis to meet assumptions of normality and homogeneity of variances.

Stepwise multiple regression (SMR) was used to select the most influential environmental factors (MAT, MAP, PET, AI, soil N, and soil P) and to estimate their contributions to NRE, PRE, and NRE:PRE. Since MAT, MAP, PET, and AI were strongly correlated to latitude (**Supplementary Figure S1**) and these parameters are of predictive and mechanistic values that were used in subsequent analysis. A partial general linear model (GLM) was applied to evaluate the relative effects of functional species groups, soil N and P, and climate (MAT, MAP, PET, and AI). The GLM separates the total variance explained by different factors into the independent effect of each factor and interactive effects of all factors (Heikkinen et al., 2005). All statistical analyses were performed using SPSS v20 (SPSS Inc., United States) and R for Window version 3.1.0 statistical software (R Core Team, 2014).

## Results

### Variations of Foliar NRE, PRE, and NRE:PRE

**FIGURE 2:**
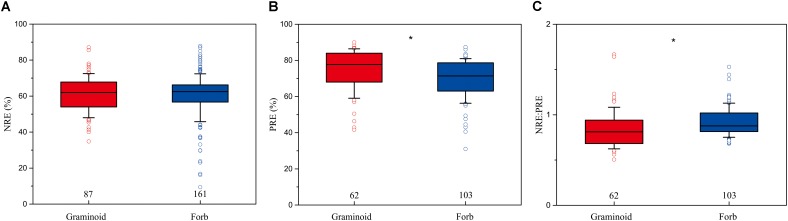
Box-whisker plots showing NRE **(A)**, PRE **(B)**, and NRE:PRE **(C)** in functional group of herbaceous species. The numbers in the figures are the sample sizes in the study sites where these two functional groups co-occurred for each group. Asterisks denote significant differences at *P* < 0.05.

The mean NRE, PRE, and NRE:PRE for all herbaceous species were 59.4% (*n* = 473, *SD* = 0.71%), 67.5% (*n* = 311, *SD* = 0.90%), and 0.89 (*n* = 309, *SD* = 0.01), respectively. PRE and NRE:PRE differed significantly between forbs and graminoids. Forbs had lower PRE (69.8%) than graminoids (74.7%) (*P* < 0.01), whereas forbs had higher NRE:PRE (0.92) compared to graminoids (0.85) (*P* < 0.05). NRE did not show significant differences between the forbs (60.2%) and graminoids (61%) (**Figure 2**).

### Climatic and Soil Influence on NRE, PRE, and NRE:PRE

For the pooled data, SMR revealed that NRE is negatively correlated with MAP and soil N (*P* < 0.01), whereas PRE is positively correlated with AI and negatively correlated with MAP and soil P (*P* < 0.05). NRE:PRE is also negatively correlated with MAP, AI, and soil N:P ratios (*P* < 0.01). MAT was excluded from all the analyses (**Table 1**).

**Table 1:** Results of stepwise multiple regression (SMR) for the effects of climatic factors and soil variables (MAT, MAP, PET, AI, soil nutrient levels and ratio) on foliar NRE, PRE, and NRE:PRE in global.

Element resorption efficiency	Adj *R*^2^ full model	Partial regression coefficient					Contribution of predictor (%)				
		MAT	MAP	PET	AI	Soil	MAT	MAP	PET	AI	Soil
**GRAMINOID**											
NRE	0.168	−0.005a	>-0.001c	–	−0.091a	>-0.002c	27.6	36.3	–	8.3	27.8
PRE	0.240	−0.011c	>−0.003c	<−0.001c	0.244c	–	39.9	29.9	15.4	14.8	–
NRE:PRE	0.251	0.012b	–	−0.003c	−0.094a	−0.027c	12.4	–	36.6	4.4	46.8
**FORB**											
NRE	0.126	0.014c	–	<−0.003c	−0.190c	–	16.4	–	17.5	66.1	–
PRE	0.178	0.019c	>−0.002c	>−0.003	0.236b	>−0.002b	18.1	20.6	23.4	20.2	17.7
NRE:PRE	0.176	–	–	>−0.001c	−0.075c	−0.004	–	–	43.2	52.4	4.4
**ALL**											
NRE	0.102	–	>−0.001c	>−0.001	–	>−0.001b	–	60.2	10.9	–	28.9
PRE	0.096	–	>−0.001c	–	0.126c	>−0.001a	–	40.4	–	30.9	31.4
NRE:PRE	0.162	–	–	>−0.001c	−0.048b	−0.011c	–	–	47.7	20.5	31.8

*a, b, and c denote significance at the 0.05, 0.01, 0.001 test level, respectively.*

The climatic factors and soil variations manifested large heterogeneity within the functional species group (**Table 1**). For example, graminoids NRE and PRE were correlated with MAT and MAP (*P* < 0.05), whereas forbs NRE was insensitive to PET (*P* > 0.05). These results show that trends in NRE, PRE, and NRE:PRE are influenced by the choice of species or regional climatic biases.

### Relative Effects of Species Group, Climate, and Soil

**FIGURE 3:**
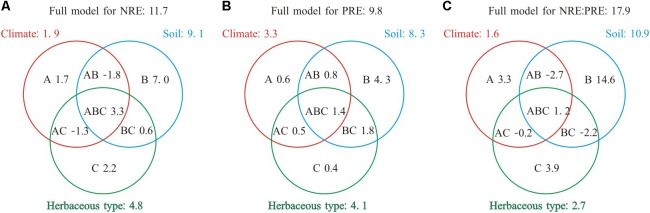
Summary of the partial general linear models (GLM) for the effects (*R*^2^, %) of climate, soil nutrient level, and species group on NRE, PRE, and NRE:PRE. A, B, and C denote the independent effects of climate, soil nutrient level, and species group, respectively, AB, AC, and BC are the interactive effects between climate and soil nutrient level, climate and species group, soil nutrient level, and herbaceous type, respectively, ABC represents the interactive effect among three different factors **(A)** Full model for NRE: 11.7; **(B)** Full model for PRE: 9.8; **(C)** Full model for NRE:PRE: 17.9.

Collectively species group, climate, and soil explained 9.8–17.9% of the variance in NRE, PRE, and NRE:PRE (**Figure 3**). The total effect of soil showed the largest contribution to the variations in NRE, PRE, and NRE:PRE (8.3–10.9%). The independent effect of soil (4.3–14.6%) was also greater than those of climate (0.6–3.3%) or species group (0.4–3.9%). Species group contributed the smallest independent variation (0.4%) to PRE. Negative values indicated suppressive interactive effects for a variable of interest (e.g., −1.3% for NRE).

## Discussion

### Functional Traits and Differences in NRE, PRE, and NRE:PRE at a Global Level

Unlike previous studies that have focused on shrub and tree species, we evaluated NRE, PRE, and NRE:PRE in the foliage of perennial herbaceous species using a global dataset. The mean values of NRE, PRE, and NRE:PRE across all species are 59.4, 67.5, and 0.89%, respectively. The values of NRE and PRE are similar to the study of Vergutz et al. (2012) (i.e., global values of 62.1 and 64.9%, respectively), but higher than those reported by Aerts (1996) based on a comparatively few data for only herbaceous species at a global scale (i.e., 50 and 57%, respectively), but only slightly lower than the values reported by Jiang et al. (2012) for 18 herbaceous species growing on the Qinghai–Tibetan Plateau (i.e., 65.2 and 67.4%). However, these values are markedly higher than those reported for woody plants by Yuan and Chen (2009) (i.e., 47 and 54%, respectively, at a global level) or by Tang et al. (2013) (i.e., 49 and 51%, respectively, at the regional scale). Thus, the nutrient resorption efficiency of herbaceous species is obviously higher than that of woody species. This has been interpreted to indicate that non-woody species are better adapted to nutrient stress than woody species by virtue of their higher internal N and P recycling (Norris and Reich, 2009; Freschet et al., 2010).

Additionally, PRE differ significantly between graminoids and forbs at a global scale, i.e., PRE are significantly higher in graminoids compared to forbs (**Figure 2B**), whereas NRE do not show this differences (**Figure 2A**). This finding is consistent with previous observations (Aerts, 1996; Jiang et al., 2012), indicating that graminoids have a competitive advantage over forbs, and also provides additional evidence that productivity, foliar nutrient allocation, and foliar biomass may lead to the higher nutrient reabsorption in graminoids than forbs (Aerts and Berendse, 1989). Furthermore, this result also supports the fact that the differentiation of P uptake serves as an important mechanism permitting the co-existence of graminoids and herbs in co-occurred regions (McCulley et al., 2004; Bertiller et al., 2005).

### Relative Influences of the Climatic and Soil in NRE, PRE, and NRE:PRE

To our knowledge, this study presents the first global-scale analyses on how nutrient resorption of N and P and their ratios vary with environmental and soil variables across a broad spectrum of herbaceous species. In addition to MAT, MAP, and soil nutrients, our analyses included PET and AI to examine the effect of drought. Contra previous studies focusing on woody species (Yuan and Chen, 2009; Reed et al., 2012; Vergutz et al., 2012), our analysis shows that MAT could not account for the variations observed in NRE, PRE, and NRE:PRE across all herbaceous species (**Table 1**). This unexpected phenomenon may result from the fact that the effects of drought on nutrient resorption overrides other abiotic factors, such as temperature. We also found that NRE:PRE were negatively correlated with PET and AI, whereas AI was positively correlated with PRE. We interpret this to indicate that PET and AI are also significant drivers of nutrient resorption efficiencies, which should be integrated into future biogeochemical cycling models.

Soil nutrient levels are also an important factor influencing nutrient resorption efficiency (Yuan et al., 2006; Yuan and Chen, 2015). Our results indicated that NRE, PRE, and NRE:PRE were negatively correlated with soil N and P levels and their ratios. This finding is consistent with most previous studies (Huang et al., 2017; Xu et al., 2017; Zhao et al., 2017) and supports the idea that plants grown in nutrient-poor environments have higher resorption capacity than those in nutrient-rich environments (Killingbeck, 1996; Wright and Westoby, 2003). Plants either acquire nutrients from the soil or from their senescing leaves (Rejmánková, 2005). Indeed, there appears to be a counterbalance between the costs of soil nutrient absorption and foliar nutrient resorption (Ratnam et al., 2008), i.e., nutrient resorption efficiency and soil nutrients content are inversely correlated.

The patterns of NRE, PRE, and NRE:PRE in relation to climate factors and soil nutrients in fact reflect the nutrient conservation strategies of herbaceous species under global climate change. The ongoing global warming and changes in rainfall regimes would be expected to result in strong heterogeneity of soil nutrient conditions and availability that vary across large temporal and spatial scales (Hedin et al., 2009). The variations in soil nutrient conditions and availability can inhibit the nutrient uptake of roots and thus constrain the metabolic activity of herbaceous species (Sun et al., 2016). Herbaceous species may increase their nutrient uptake through improving NRE and PRE, thus reducing their dependence on the supply of soil nutrients (e.g., rapid growth, high leaf nutrient contents, and an accelerated life history; Adler et al., 2014). This strategy can collectively reduce N and P acquisition by roots and their associated ectomycorrhiza (Lambers et al., 2008). Therefore, the acclimation responses of herbaceous species to variation of soil nutrient availability may contribute to these apparent patterns. Climate, soil nutrient levels, and species groups collectively affect the biogeography of plant nutrient resorption efficiency in complex ways. In this study, partial GLM regression was used to examine the independent effects of each factor and their interactive effects (Heikkinen et al., 2005). We found that the independent effect of soil nutrient level was the largest contributor to observed variations in foliar NRE, PRE, and NRE:PRE (**Figure 3**), suggesting that soil nutrient level exerts a dominant control of NRE, PRE, and NRE:PRE, while climate and herbaceous types had relatively smaller effects on NRE, PRE, and NRE:PRE. This supposition is consistent with most of previous studies (Aerts, 1996; Aerts and Chapin, 1999; Yuan and Chen, 2015). However, we should note that total soil nutrient level may not be indicative of soil nutrient availability though it has been widely used in many ecological studies (Rejmánková, 2005; Zhang et al., 2018). Factors such as soil pH, humus, and mineralization may also influence soil nutrient cycling and bioavailability, leading to the un-relatedness between these two variables (Barber, 1984; Vitousek, 1984; Yuan et al., 2006). For example, Fe in higher pH soils is easily oxidized and therefore becomes unavailable for plants (Morrissey and Guerinot, 2009). Nevertheless, total soil nutrient status can be considered as a preliminary variable to investigate the relative importance of climate and soil on plant nutrient resorption efficiency at large (e.g., global) scale (Yuan and Chen, 2009). Due to the lack of supporting data of soil nutrient availability for each site in our dataset, we are unable to analyze the influence of soil nutrient availability on NRE, PRE, and NRE:PRE. Plant nutrient resorption is affected by soil nutrient availability (Oleksyn et al., 2003; Yuan et al., 2005a), hence, extensive data of soil nutrient availability with supporting biogeographic information at global scale are urgently needed to provide more direct evidence for the effect of soil nutrient availability on NRE, PRE, and NRE:PRE.

### Species Group Differences in Foliar NRE, PRE, and NRE:PRE

It is generally assumed that the functional traits of different plant groups will converge across increasing geographic scales (Niklas et al., 2007; Santiago and Wright, 2007). However, our analyses at the global level reveal large differences in foliar NRE, PRE, and NRE:PRE within functional species groups (**Table 1**). For example, forbs NRE and NRE:PRE were not correlated with MAP, whereas graminoids NRE was correlated with MAP. These results may reflect the influence of soil nutrient availability on different conservation strategies including nutrient resorption (Oleksyn et al., 2003; Yuan et al., 2005b; Adler et al., 2014).

## Conclusion

Our analyses indicate that, when viewed at a worldwide level, more than half of all foliar N and P is resorbed during foliage senescence in perennial herbaceous species and that NRE, PRE and their ratios differ significantly from those of woody species. Our analyses additionally indicate that soil nutrient levels are the dominant factor in predicting NRE, PRE, and NRE:PRE across herbaceous species and manifest discernable and significant biogeographic patterns. Our results will improve the understanding of variations in N and P resorption and predict the responses of plant community to global climate change. Our findings also indicate that the PET and AI are additional important abiotic factors and should be integrated into future biogeochemical models to predict potential changes in ecosystem dynamics in response to changing climate and attempts to model biogeochemical cycling at a global scale.

## Author Contributions

ZW, HH, and JR designed the research. ZW, ZF, QZ, and MW collected the data. ZW, ZF, and HH analyzed the data. ZW, ZF, QZ, JR, HH, and KN wrote the manuscript and all authors gave final approval for publication.

## Conflict of Interest Statement

The authors declare that the research was conducted in the absence of any commercial or financial relationships that could be construed as a potential conflict of interest.

## Footnotes

1 http://www.fallingrain.com/world/

2 http://www.worldclim.org/
