# Supplementary material for: Global Data Analysis Shows That Soil Nutrient Levels Dominate Foliar Nutrient Resorption Efficiency in Herbaceous Species
Source: Front Plant Sci. 2018 Sep 26;9:1431. doi: 10.3389/fpls.2018.01431 (PMC6168711; doi:10.3389/fpls.2018.01431)
Supplement: Supplementary file 1 [file Table_1.DOCX]

Literature Cited

1. Aerts R, Cornelissen JHC, van Logtestijn REP, Callaghan TV. 2007. Climate change has only a minor impact on nutrient resorption parameters in a high-latitude peatland. Oecologia 151:132-139.
2. Allison SD, Vitousek PM. 2004. Rapid nutrient cycling in leaf litter from invasive plants in Hawai'i. Oecologia 141:612-619.
3. Bertiller MB, Sain CL, Carrera AL, Vargas DN. 2005. Patterns of nitrogen and phosphorus conservation in dominant perennial grasses and shrubs across an aridity gradient in Patagonia, Argentina. Journal of Arid Environments 62:209-223.
4. Bilgin A, Yalcin E, Kutbay HG, Kok T. 2004. Foliar N and P dynamics of Heracleum platytaenium (Apiaceae) in relation to edaphic charcteristics along an elevation gradient in northern Turkey. Annales Botanici Fennici 41:85-93.
5. Bowman WD, Theodose TA, Fisk MC. 1995. Physiological and production responses of plant growth forms to increases in limiting resources in alpine tundra: implications for differential community response to environmental change. Oecologia 101:217-227.
6. Cakir YB, Ozbucak T, Kutaby HG, Kilic D, Bilgin A, Huseyinova R. 2010. Nitrogen and phosphorus resorption in a salt marsh in northern Turkey. Turkey Journal of Botany 34:311-322.
7. Campanella MV, Bertiller MB. 2011. Is N-resorption efficiency realted to secondary compounds and leaf longevity in coexisting plant species of the arid Patagonian Monte, Argentina? Austral Ecology 36:395-402.
8. Carrera AL, Sain CL, Bertiller MB. 2000. Pattern of nitrogen conservation in shurbs and grasses in the Ptagonian Monte, Argentina. Plant Soil 224:185-193.
9. Cartaxana P, Catarino F. 2002. Nitrogen resorption from senescing leaves of three salt marsh plant species. Plant Ecology 159:95-102.
10. Chapin FS, Shaver GR. 1989. Differences in growth and nutrient use among Arctic plant growth forms. Functional Ecology 3:73-80.
11. Demars BG, Boerner REJ. 1997. Foliar Phosphorus and Nitrogen Resorption in Three Woodland Herbs of Contrasting Phenology. Castanea 62:43-54.
12. Distel RA, Moretto AS, Didone NG. 2003. Nutrient resorption from senescing leaves in two stipa species native to central Argentina. Austral Ecology 28:210-215.
13. Dordas CA, Sioulas C. 2009. Dry matter and nitrogen accumulation, partitioning, and retranslocation in safflower (Carthamus tinctorius L.) as affected by nitrogen. Field Crops Research 110:35-43.
14. Freschet GT, Cornelissen JHC, van Logtestijn RSP, Aerts R. 2010. Substantial nutrient resorption from leaves, stems and roots in a subarctic flora: what is the link with other resource economics traits? New Phytologist 186:879-889.
15. Guo C, Cai JY, Qi J, Zhou HY, Li M, Hu QW. 2016. Nitrogen and phosphorus resorption of six dominant plant species in Poyang lake wetlands. Chinese Journal of Ecology 35:692-697.
16. Gursoy S, Kutbay HG, Kilic DD, Huseyinova R, Bilgin A, Yilmaz H. 2013. Nitrogen and phosphorus resorption in two wetland macrophytes. Parkistan Journal of Botany 45:1707-1714.
17. Gusewell S. 2005. Nutrient resorption of wetland graminoids is related to the type of nutrient limitation. Functional Ecology 19:344-354.
18. Huang JJ, Boerner REJ. 2007. Effects of fire alone or combined with thinning on tissue nutrient concentrations and nutrient resorption in Desmodium nudiflorum. Oecologia 153:233-243.
19. Huang JY, Yu HL, Wang B, Li LH, Xiao GJ, Yuan ZY. 2012. Nutrient resorption based on different estimations of five perennial herbaceous species from the grassland in inner Mongolia, China. Journal of Arid Environments 76:1-8.
20. Hobbie S, Gough L. 2002. Foliar and soil nutrients in tundra on glacial landscapes of contrasting ages in northern Alaska. Oecologia 131:453-462.
21. Jach-Smith LC, Jackson RD. 2015. Nitrogen conservation decreases with fertilizer addition in two perennial grass cropping systems for bioenergy. Agriculture, Ecosystems and Environment 204:62-71.
22. Jiang CM, Yu GR, Li YN, Cao GM, Yang ZP, Sheng WP, Yu WT. 2012. Nutrient resorption of coexistence species in alpine meadow of the Qinghai-Tibetan Plaeau explain plant adaptation to nutrient-poor. Ecological Engineering 44:1-9.
23. Kazakou E, Garnier E, Navas ML, Roumet C, Collin C, Laurent G. 2007. Components of nutrient residence time and the leaf economics spectrum in species from Mediterranean old-fields differing in successional status. Functional Ecology 21:235-245.
24. Lawniczak AE. Nitrogen, phosphorus, and Potassium resorption efficiency and proficiency of four emergent Macrophytes from nutrient-rich wetlands. 2011. Polish Journal of Environmental Studies 20:1227-1234.
25. Li L, Gao XP, Li XY, Lin LS, Zeng FJ, Gui DW, Lu Y. 2016. Nitrogen (N) and phosphorus (P) resorption of two dominant alpine perennial grass species in response to contrasting N and P availability. Environmental and Experimental botany 127:37-44.
26. Li LJ. Zeng DH, Mao R, Yu ZY. 2012. Nitrogen and phosphorus resorption of Artemisia scoparia, Chenopodium acuminatum, Cannabis sativa, and Phragmites communis under nitrogen and phosphorus additions in a semi-arid grassland, China. 2012. Plant soil Environment 58:446-451.
27. Li X, Liu J, Fan J, Ma Y, Ding S, Zhong Z, Wang D. 2015. Combined effects of nitrogen addition and litter manipulation on nutrient resorption of Leymus chinensis in a semi-arid grassland of northern China. Plant Biology 17:9-15.
28. Li YH, Han GD, Wang Z, Wang ZW, Zhao ML, Wang SRN. 2015. Nitrogen resorption efficiency of perennial plant decreases with warming and nitrogen addition in desert steppe. Acta Ecologica Sinica 35:5948-5956.
29. Li YL, Cui D, Chen J, Mao W, Zhao XY. 2013. Divergence of nitrogen resorption efficiency of dominant perennial plants in Horqin sandy grassland. Journal of Desert Research 33:688-695.
30. Liang DF, Zhang JJ, Zhang ST. 2015. Patterns of nitrogen resorption in functional groups in a Tibetan alpine meadow. Folia Geobotanica 50:267-274.
31. Liu CC, Liu YG, Guo K, Wang SJ, Yang Y. 2014 Concentrations and resorption patterns of 13 nutrients in different plant functional types in the karst region of south-western China. Annals of Botany 113:873-885.
32. Lv XT, HanXG. Nutrient resorption responses to water and nitrogen amendment in semi-arid grassland of Inner Mongolia, China. 2010. 327:481-491.
33. Lv XT, Cui Q, Wang QB, Han XG. 2011. Nutrient resorption response to fire and nitrogen addition in a semi-arid grassland. Ecological Engineering 37:534-538.
34. Lv XT, Reed SC, Yu Q, Han XG. 2016. Nutrient resorption helps drive intra-specific coupling of foliar nitrogen and phosphorus under nutrient-enriched conditions. Plant Soil 398:111-120.
35. Mao R, Song CC, Zhang XH, Wang XW, Zhang ZH. 2013. Response of leaf, sheath and stem nutrient resorption to 7 years of N addition in freshwater wetland. Plant Soil 364:385-394.
36. Mao R, Zeng DH, Zhang XH, Song CC. 2015. Response of plant nutrient resorption to phosphorus addition in freshwater marsh of Northeast China. Scientific Reports 5:8097.
37. Mao W, Li Y, Cui J, Zuo X, Zhao X. 2011. Variations in folia nutrient resorption eficiency of different plant growth forms in a temperate sandy grassland. Polish Journal of Ecology 59:355-365.
38. Pastor-Pastor A, Gonzalez-Paleo L, Vilela A, Ravetta D. 2015. Age-related changes in nitrogen resorption and use efﬁciency in the perennial new crop Physaria mendocina (Brassicaceae). Industrial Crops and Products. 65:227-232.
39. Peri PL, Lasagno RG. 2010. Biomass, carbon and nutrient storage for dominant grasses of cold temperate steppe grasslands in southern Patagonia, Argentina. Journal of Arid Environments 74:23-34.
40. Quested H, Press M, Callaghan T, Cornelissen H. 2002. The hemiparasitic angiosperm Bartsia alpina has the potential to accelerate decomposition in sub-arctic communities. Oecologia 130:88-95.
41. Ratnam J, Sankaran M, Hanan NP, Grant RC, Zambatis N. 2008. Nutrient resorption patterns of plant functional groups in a tropical savanna: variation and functional significance. Oecologia 157:141-151.
42. Ren LY, Yuan ZY, Wang HY, Wen ZJ. 2005. Nitrogen resorption of three life-forms (trees, shrubs and grasses) in the semi-arid region of North China. Acta Botany Boreal.-Occident. Sinica 25:497-502.
43. Richardson SJ, Allen RB, Doherty JE. 2008. Shifts in leaf N : P ratio during resorption reflect soil P in temperate rainforest. Functional Ecology 22:738-745.
44. Shaver GR, Stuart Chapin F. 1991. Production: biomass relationships and element cycling in contrasting arctic vegetation types. Ecological Monographs 61:1-31.
45. Wang ZN, Lu JY, Yang HM, Zhang X, Luo CL, Zhao YX. 2014. Resorption of nitrogen, phosphorus and potassium from leaves of Lucerne stands of different ages. Plant Soil 383:301-312.
46. Yuan ZY, Li LH, Han XG, Huang JH, Jiang GM, Wan SQ, Zhang WH, Chen QS. 2005. Nitrogen resorption from senescing leaves in 28 plant species in a semi-arid region of northern China. Journal of Arid Environments 63:19-202.
47. Yuan, ZY, Li LH, Han XG, Huang JH, Jiang GM, Wan SQ. 2005. Soil characteristics and nitrogen resorption in Stipa krylovii native to northern China. Plant and Soil 273:257-268.
48. Zhao GS, Xiong DP, Shi PL, Feng YF, Wu JS, Zhang XF, Zeng ZX. 2016. Leaf nitrogen resorption efficiency of Stipa purpurea and its deteminants along a precipitation gradient on the Changtang Plateau. Acta Ecologica Sinica 36:3419-3428.
49. Zhao GS, Shi PL, Wu JS, Xiong DP, Zong N, Zhang XZ. 2017. Foliar nutrient resorption patterns of four functional plants along a precipitation gradient on the Tibetan Changtang Plateau. Ecology Evolution 00:1-12.
50. Zotz G. 2004. The resorption of phosphorus is greater than that of nitrogen in senescing leaves of vascular epiphytes from lowland Panama. Journal of Tropical Ecology 20:693-696.

Table S1. A list of all perennial herbaceous species complied by this study. G = graminoid species, F = forb species, P = perennial species.

| Species | Family | Altitude (m) | G/F |
| --- | --- | --- | --- |
| *Achnatherum sibiricum* | Gramineae | 1250 | G |
| *Acomastylis rosii* | Rosaceae | 3520 | F |
| *Agropyron cristatum* | Gramineae | 1380 | G |
| *Agropyrum smithii* | Gramineae | 115 | G |
| *Anahalis lactea* | Compositae | 3500 | F |
| *Andropogon gerardii* | Gramineae | 319 | G |
| *Anemone rivularis* | [Ranunculaceae](http://frps.eflora.cn/frps/Ranunculaceae) | 3500 | F |
| *Anemone trullifolia* | [Ranunculaceae](http://frps.eflora.cn/frps/Ranunculaceae) | 3500 | F |
| *Angelica archangelica* | [Apiaceae](https://en.wikipedia.org/wiki/Apiaceae) | 375 | F |
| *Anthoxanthum odoratum* | Gramineae | 1 | G |
| *Anthurium brownii* | Compositae | 140 | F |
| *Anthurium clavigerum* | Compositae | 140 | F |
| *Anthurium friedrichsthalii* | Compositae | 140 | F |
| *Anthurium scandens* | Compositae | 140 | F |
| *Arenaria serpyllifolia* | Caryophyllaceae | 60 | F |
| *Artemesia scopulorum* | Compositae | 3520 | F |
| *Artemisia frigida* | Compositae | 1324 | F |
| *Artemisia halodendron* | Compositae | 500 | F |
| *Artemisia lavandulaefolia* | Compositae | 360 | F |
| *Artemisia mongolica* | Compositae | 3500 | F |
| *Artemisia santonicum* | Compositae | 25 | F |
| *Artemisia scoparia* | Compositae | 259 | F |
| *Artemisia sieversiana* | Compositae | 1380 | F |
| *Arthraxon* hispidus | Gramineae | 1311 | G |
| *Arthrocnemum perenne* | Chenopodiaceae | 0 | F |
| *Aspasia principissa* | Orchidaceae | 140 | F |
| *Aster ageratoides* | Compositae | 1311 | F |
| *Aster diplostephioides* | Compositae | 3500 | F |
| *Aster flaccidus* | Compositae | 3250 | F |
| *Bartsia alpina* | Orobanchaceae | 360 | F |
| *Bistorta bistortoides* | Polygonaceae | 3520 | F |
| *Bistorta vivipara* | Polygonaceae | 3520 | F |
| *Boumus setifolius* | Gramineae | 115 | G |
| *Bouteloa curtipendula* | Gramineae | 115 | G |
| *Brachypodium phoenicoides* | Gramineae | 60 | G |
| *Bromus erectus* | Gramineae | 60 | G |
| *Bromus madnritensis* | Gramineae | 60 | G |
| *Bromus setifolius* | Gramineae | 100 | G |
| *Buchloe dactyloides* | Gramineae | 115 | G |
| *Calamagrositis epigeios* | Gramineae | 360 | G |
| *Calamagrositis preudophragmites* | Gramineae | 360 | G |
| *Calamagrostis canadensis* | Gramineae | 760 | G |
| *Calamagrostis epigejos* | Gramineae | 1380 | G |
| *Calamagrostis lapponica* | Gramineae | 377 | G |
| *Calamagrostis purpurascans* | Gramineae | 3520 | G |
| *Calamintha nepeta* | Lamiaceae | 60 | F |
| *Cannabis sativa* | [Moraceae](http://frps.eflora.cn/frps/Moraceae) | 259 | F |
| *Cardamine concatenata* | Brassicaceae | 271 | F |
| *Carex acutiformis* | Cyperaceae | 490 | G |
| *Carex alrofusca* | Cyperaceae | 3250 | G |
| *Carex andina* | Cyperaceae | 453 | G |
| *Carex aquatilis* | Cyperaceae | 760 | G |
| *Carex bigelowii* | Cyperaceae | 760 | G |
| *Carex capitata* | Cyperaceae | 360 | G |
| *Carex curta* | Cyperaceae | 1 | G |
| *Carex diandra* | Cyperaceae | 1 | G |
| *Carex duriuscula* | Cyperaceae | 360 | G |
| *Carex elata* | Cyperaceae | 490 | G |
| *Carex enervis* | Cyperaceae | 3500 | G |
| *Carex flacca* | Cyperaceae | 490 | G |
| *Carex korshinskyi* | Cyperaceae | 1380 | G |
| *Carex* lanceolata | Cyperaceae | 1311 | G |
| *Carex panicea* | Cyperaceae | 490 | G |
| *Carex rostrata* | Cyperaceae | 375 | G |
| *Carex rupestris* | Cyperaceae | 3520 | G |
| *Carex vaginata* | Cyperaceae | 360 | G |
| *Carthamus tinctorius L* | Compositae | 40 | F |
| *Catasetum viridiflavum* | Orchidaceae | 140 | F |
| *Caularthron bilamellatum* | Orchidaceae | 140 | F |
| *Ceratoides arborescens* | Chenopodiaceae | 1380 | F |
| *Comarum palustre* | Rosaceae | 375 | F |
| *Convolvulus ammannii* | Convolvulaceae | 1456 | F |
| *Convolvulus arvensis* | Convolvulaceae | 360 | F |
| *Cornus suecica* | Cornaceae | 375 | F |
| *Crepis foetida* | Compositae | 60 | F |
| *Dacty glomerata* | Gramineae | 60 | G |
| *Daucus carota* | Apiaceae | 60 | F |
| *Delphinium kamaonense* | [Ranunculaceae](http://frps.eflora.cn/frps/Ranunculaceae) | 3500 | F |
| *Deschampsia caespitosa* | Gramineae | 3500 | G |
| *Deschampsia flexuosa* | Gramineae | 375 | G |
| *Deyeuxia angustifolia* | Gramineae | 56 | G |
| *Dimerandra emarginata* | Orchidaceae | 140 | F |
| *Elsholtzia* rugulosa | Lamiaceae | 1311 | F |
| *Elymus nutans* | Gramineae | 3250 | G |
| *Encyclia chimborazoensis* | Orchidaceae | 140 | F |
| *Ephedra sinica* | Ephedraceae | 360 | F |
| *Epidendrum imatophyllum* | Orchidaceae | 140 | F |
| *Epidendrum nocturnum* | Orchidaceae | 140 | F |
| *Epidendrum rigidum* | Orchidaceae | 140 | F |
| *Epilobium angustifolium* | [Onagraceae](http://frps.eflora.cn/frps/Onagraceae) | 375 | F |
| *Epilobium latifolium* | Onagraceae | 760 | F |
| *Eriophorum vaginatum* | Cyperaceae | 760 | G |
| *Euphrasia frigida* | Orobanchaceae | 360 | F |
| *Ferula bungeana* | Umbelliferae | 360 | F |
| *Festuca ovina* | Gramineae | 3250 | G |
| *Festuca pallescens* | Gramineae | 700 | G |
| *Filipendula ulmaria* | Rosaceae | 375 | F |
| *Gentiana macrophylla* | [Gentianaceae](http://frps.eflora.cn/frps/Gentianaceae) | 3500 | F |
| *Gentiana straminea* | [Gentianaceae](http://frps.eflora.cn/frps/Gentianaceae) | 3250 | F |
| *Gentianopsis barbata* | [Gentianaceae](http://frps.eflora.cn/frps/Gentianaceae) | 3500 | F |
| *Geranium rotundifolium* | Geraniaceae | 60 | F |
| *Geranium sylvaticum* | Geraniaceae | 375 | F |
| *Geranium wilfordii* | Geraniaceae | 3500 | F |
| *Glyceria maxima* | Gramineae | 75 | G |
| *Glyceria spiculosa* | Gramineae | 56 | G |
| *Gongora quinquenervis* | Orchidaceae | 140 | F |
| *Hedychium gardnerianum* | Zingiberaceae | 150 | F |
| *Helianthus annuus* | Compositae | 1380 | F |
| *Helianthus grosseserratus* | Compositae | 319 | F |
| *Heracleum platytaenium* | Apiaceae | 30 | F |
| *Heteropogon contortus* | Gramineae | 1311 | G |
| *Hilaria jamesii* | Gramineae | 115 | G |
| *Hordeum comosum* | Gramineae | 115 | G |
| *Incarvillea* *arguta* | Bignoniaceae | 1311 | F |
| *Indocalamus tessellates* | Gramineae | 50 | G |
| *Iris* *tectorum* | Iridaceae | 1311 | F |
| *Jarava speciosa* | Gramineae | 99 | G |
| *Kobresia capillifolia* | Gramineae | 3500 | G |
| *Kobresia humulis* | Cyperaceae | 3250 | G |
| *Kobresia myosuroides* | Cyperaceae | 3520 | G |
| *Kobresia setchwanensis* | Gramineae | 3500 | G |
| *Koeleria cristata* | Gramineae | 3250 | G |
| *Koeleria glauca* | Gramineae | 3500 | G |
| *Lamiophlomis rotata* | Labiatae | 3500 | F |
| *Lancea tibetica* | Scrophulariaceae | 3250 | F |
| *Leontopodium* *leontopodioides* | Compositae | 1311 | F |
| *Leymus chinensis* | Gramineae | 360 | G |
| *Leymus secalinus* | Gramineae | 360 | G |
| *Ligularia virgaurea* | Compositae | 3250 | F |
| *Linum perenne* | Linaceae | 1380 | F |
| *Messerschmidia sibirica* | Boraginaceae | 360 | F |
| *Miscanthus* *floridulus* | Gramineae | 1311 | G |
| *Molinia caerulea* | Gramineae | 490 | G |
| *Morina chinensis* | Dipsacaceae | 3250 | F |
| *Nassella tenuis* | Gramineae | 99 | G |
| *Oncidium ampliatum* | Orchidaceae | 140 | F |
| *Oplismenus* *undulatifolius* | Gramineae | 1311 | G |
| *Otanthus maritimus* | Compositae | 25 | F |
| *Paspalum dilatatum* | Gramineae | 115 | G |
| *Pedicularis hirsuta* | Scrophulariaceae | 360 | F |
| *Pedicularis kansuensis* | Scrophulariaceae | 3500 | F |
| *Pedicularis lapponica* | Scrophulariaceae | 360 | F |
| *Pedicularis sceptrum-carolinum* | Scrophulariaceae | 360 | F |
| *Pedicularis semitorta* | Scrophulariaceae | 3500 | F |
| *Pedicularis szetschuanica* | Scrophulariaceae | 3500 | F |
| *Peperomia macrostachya* | Piperaceae | 140 | F |
| *Petasites frigidus* | Compositae | 760 | F |
| *Phragmites australis* | Gramineae | 360 | G |
| *Phragmites communis* | Gramineae | 259 | G |
| *Physaria mendocina* | Brassicaceae | 390 | F |
| *Picris hieraciodies* | Compositae | 60 | F |
| *Poa dusenii* | Gramineae | 316 | G |
| *Poa ligularis* | Gramineae | 99 | G |
| *Poa orinosa* | Gramineae | 3250 | G |
| *Poa pachyantha* | Gramineae | 3500 | G |
| *Polygonum bistorta* | Polygonaceae | 760 | F |
| *Polygonum divaricatum* | Polygonaceae | 1380 | F |
| *Polygonum viviparum* | Polygonaceae | 3500 | F |
| *Polystachia foliosa* | Orchidaceae | 140 | F |
| *Potentilla acaulis* | Rosaceae | 1324 | F |
| *Potentilla anserina* | Rosaceae | 3250 | F |
| *Potentilla bifurca* | Rosaceae | 1250 | F |
| *Potentilla* *chinensis* | Rosaceae | 1311 | F |
| *Potentilla fisch* | Rosaceae | 360 | F |
| *Potentilla fragarioides* | Rosaceae | 3500 | F |
| *Potentilla nivea* | Rosaceae | 3250 | F |
| *Potentilla tanacetifolia* | Rosaceae | 1450 | F |
| *Rubia peregrina* | Rubiaceae | 60 | F |
| *Rubus chamaemorus* | Rosaceae | 377 | F |
| *Rubus hispidus* | Rosaceae | 70 | F |
| *Rubus saxatilis* | Rosaceae | 375 | F |
| *Salicornia prostrata* | Chenopodiaceae | 25 | F |
| *Sanguisorba officinalis* | Rosaceae | 1450 | F |
| *Saposhnikovia divaricata* | Apiaceae | 60 | F |
| *Sarracenia purpurea* | Sarraceniaceae | 70 | F |
| *Sasa kurilensis* | Gramineae | 300 | G |
| *Saussurea alpina* | Compositae | 375 | F |
| *Saussurea lanuginosa* | Compositae | 3500 | F |
| *Saussurea nigrescens* | Compositae | 3250 | F |
| *Saussurea pachyneura* | Compositae | 3500 | F |
| *Saussurea stella* | Compositae | 3500 | F |
| *Saussurea superba* | Compositae | 3250 | F |
| *Schizachyrium scoparium* | Gramineae | 115 | G |
| *schoenoplectus lacustris* | Cyperaceae | 0 | G |
| *Scirpus distigmaticus* | Cyperaceae | 3250 | G |
| *Scirpus pumilus* | Gramineae | 3500 | G |
| *Scutellaria baicalensis* | Labiatae | 3500 | F |
| *Senecio* *scandens* | Compositae | 1311 | F |
| *Seriphidium rhodanthum* | Gramineae | 3100 | G |
| *Setaria palmifolia* | Gramineae | 150 | G |
| *Smilacina racemosa* | Liliaceae | 271 | F |
| *Sobralia suaveolens* | Orchidaceae | 140 | F |
| *Soghastrum nutans* | Gramineae | 319 | G |
| *Spartina maritima* | Gramineae | 0 | G |
| *Spergularia marina* | Caryophyllaceae | 25 | F |
| *Stipa aliena* | Gramineae | 3250 | G |
| *Stipa brachychaeta* | Gramineae | 141 | G |
| *Stipa breviflora* | Gramineae | 1456 | G |
| *Stipa capillata* | Gramineae | 3100 | G |
| *Stipa chrysophylla* | Gramineae | 120 | G |
| *Stipa comata* | Gramineae | 115 | G |
| *Stipa grandis* | Gramineae | 1250 | G |
| *Stipa gynerioides* | Gramineae | 141 | G |
| *Stipa humilis* | Gramineae | 115 | G |
| *Stipa krylovii* | Gramineae | 1380 | G |
| *Stipa purpurea* | Gramineae | 4756 | G |
| *Stipa speciosa* | Gramineae | 115 | G |
| *Stipa tenuis* | Gramineae | 115 | G |
| *Stipa viridula* | Gramineae | 115 | G |
| *Taraxacum mongolicum* | Compositae | 3500 | F |
| *Teucrium chamaedrys* | Lamiaceae | 60 | F |
| *Teucrium* *quadrifarium* | Lamiaceae | 1311 | F |
| *Thalictrum alpinum* | [Ranunculaceae](http://frps.eflora.cn/frps/Ranunculaceae) | 3500 | F |
| *Thalictrum aquilegifolium* | Ranunculaceas | 360 | F |
| *Thalictrum squarrosum* | Ranunculaceae | 1450 | F |
| *Themeda* japonica | Gramineae | 1311 | G |
| *Tordylium maximum* | Apiaceae | 60 | F |
| *Trientalis europaea* | Primulaceae | 375 | F |
| *Trigonidium egertonianum* | Orchidaceae | 140 | F |
| *Trillium flexipes* | Liliaceae | 271 | F |
| *Tripolium vulgare* | Compositae | 360 | F |
| *Trollius europaeus* | [Ranunculaceae](https://en.wikipedia.org/wiki/Ranunculaceae) | 375 | F |
| *Trollius farreri* | [Ranunculaceae](http://frps.eflora.cn/frps/Ranunculaceae) | 3500 | F |
| *Typha angustifolia* | Typhaceae | 75 | G |
| *Typha latifolia* | Typhaceae | 0 | G |
| *Veronica eriogyne* | Scrophulariaceae | 3500 | F |
| *Veronica persica* | Scrohulariaceae | 60 | F |


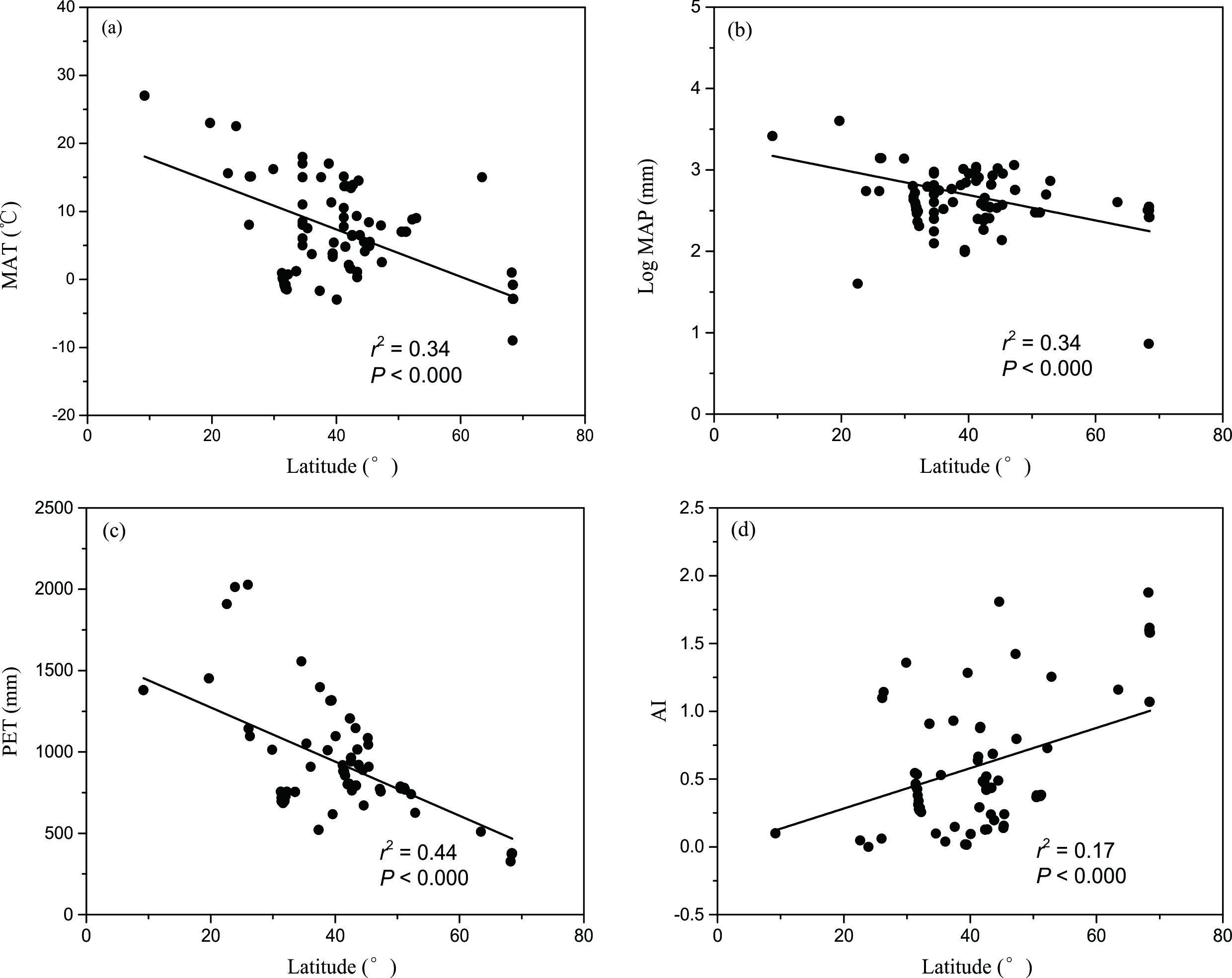
Figure S1.Variations in MAT (a), MAP (b), PET (c) and AI (d) along latitudinal gradients.
